# Checklist of Mosses of Uzbekistan

**DOI:** 10.3390/plants15213389

**Published:** 2026-11-01

**Authors:** Farrukh Abdirasulov, Zhalov Kholmurod, Marko S. Sabovljević

**Affiliations:** 1 Department of Botany, Sharof Rashidov Samarkand State University, University Boulevard 15, Samarkand UZ-140104, Uzbekistan; 2 Institute of Botany and Botanical Garden, Faculty of Biology, University of Belgrade, Takovska 43, RS-11000 Belgrade, Serbia; 3 Department of Plant Biology, Institute of Biology and Ecology, Faculty of Science, Pavol Jozef Šafárik University in Košice, Mánesova 23, SK-040 01 Košice, Slovakia

**Keywords:** bryophytes, Bryophyta, survey, inventory, Uzbekistan, Central Asia, diversity, biogeography, taxonomy

## Abstract

The present checklist documents 241 moss taxa recorded from Uzbekistan, including 234 species, one subspecies, five varieties, and one form. Among them, 43 additional taxa were reported for the country. The checklist is based on a comprehensive critical revision of the published literature, herbarium collections, and recent taxonomic treatments. Historical records and synonymy were carefully re-evaluated in accordance with current international nomenclatural standards and modern bryological classifications. Out of 234 species, 78 have not been recorded for more than two decades. This updated synthesis provides a reliable and standardized account of the moss flora of Uzbekistan, correcting previous taxonomic inconsistencies and integrating recent floristic and distributional data. The results significantly enhance the understanding of bryophyte diversity in Central Asia and establish a solid baseline for future taxonomic, ecological, biogeographical, and conservation studies in the region.

## 1. Introduction

Central Asia is one of the most heterogeneous floristic regions of the Eurasian continental area, characterized by a complex geological history, pronounced altitudinal gradients, and a wide spectrum of ecological conditions ranging from arid deserts to high mountain ecosystems [1]. In addition, the “Mountains of Central Asia” rank among one of the most poorly studied biodiversity hotspots [2]. Such a plethora of environmental diversity allows all prerequisites to have comparatively rich bryophyte flora, which includes a significant number of regionally endemic and biogeographically important taxa.

Despite more than a century of bryological exploration in the region, bryophyte diversity remains unevenly documented across Central Asia, since most of the prior investigations were not fully intentional or targeted but rather more incidental. Uzbekistan, a landlocked country in Central Asia, in particular, remains comparatively underexplored, with existing data on its bryoflora fragmented across the historical literature, regional floristic studies, and herbarium-based records worldwide. Previous studies from neighboring countries such as Tajikistan, Kazakhstan, and Kyrgyzstan demonstrate a more complete floristic assessment, whereas Uzbekistan still lacks a comprehensive national synthesis.

The increasing availability of herbarium data, digitized biodiversity databases, and the historical taxonomic literature provides an opportunity to critically reassess bryophyte diversity in the region. However, taxonomic inconsistencies, synonymy issues, and incomplete geographical coverage continue to limit a unified understanding of moss diversity in Central Asia.

Therefore, a comprehensive and critically revised checklist is essential to integrate historical records with modern data sources and to provide a reliable update on Uzbek moss survey for future taxonomic, ecological, and conservation studies. All records published up to the end of 2025 were included in the present study, with the aim to document the first critical list on moss flora diversity in Uzbekistan, as well as to highlight the old and unreliable records.

Central Asia comprises one of the most important dryland regions in the world, accounting for nearly one-tenth of the global dryland area [3], with Uzbekistan being a home to a significant proportion of the regional biodiversity. Within the region, special emphasis on central Asian mountain areas, one of the world biodiversity hotspots, considered to be the most vulnerable, yet insufficiently studied, should be given [2]. In the north, Uzbekistan borders Kazakhstan; in the east and southeast, Kyrgyzstan and Tajikistan; in the west, Turkmenistan; and in the south, Afghanistan [4]. Uzbekistan occupies the central part of Central Asia, and the country’s relief is highly diverse, comprising two contrasting geomorphological zones: extensive lowland plains and complex mountain systems [1]. The principal mountain systems of Uzbekistan are the Western Tian Shan and the Pamir-Alai, represented within the country only by their marginal ranges, while the majority of the major mountain ridges are shared with neighboring states. The elevational gradient across the region is pronounced, ranging from 12 m below sea level at the Sarygamysh Lake depression to 4643 m above sea level at the summit of Khazret Sultan [4,5]. In the botanical–geographical literature, the following altitudinal zonation is adopted for the territory of Uzbekistan and Central Asia: desert zone (20–500 m a.s.l.), foothill zone (500–1500 m a.s.l.), mountain zone (1500–2600 m a.s.l.; referred to as the low-mountain belt in Russian-language sources), subalpine zone (2600–3200 m a.s.l.; middle-mountain belt), and alpine zone (3200–4600 m a.s.l.; high-mountain belt) [5]. Approximately 79% of the territory is occupied by arid and semi-arid plains, topographically dominated by the vast Kyzylkum Desert and the Ustyurt Plateau [3]. According to the Köppen climate classification, Uzbekistan’s territory falls within the cold desert, cold semi-arid, and humid continental climate zones [6]. The climate of Uzbekistan is predominantly continental and arid, characterized by wide diurnal and seasonal temperature fluctuations [4]. Annual precipitation ranges from 70 to 100 mm on the plains of the country’s northwestern part to as much as 1200 mm in mountainous areas, with more than 70% of precipitation occurring during the autumn–spring period [4].

## 2. Results

### 2.1. Historical Overview of Bryological Studies in Central Asia with Special Emphasis to Uzbekistan

The first studies of bryophytes in Central Asia date back to the second half of the nineteenth century and are closely linked to large-scale botanical explorations of Turkestan. One of the earliest collectors of bryological material in the region was A. O. Fedchenko, whose expeditions initiated the first systematic investigations of the Central Asian bryoflora [7]. Between 1870 and 1900, extensive botanical expeditions were carried out across the Pamir-Alay, Tien Shan, and Kopetdag mountain systems, as well as the Kyzylkum and Karakum deserts. These expeditions involved prominent botanists such as E. Regel, V. L. Komarov, V. I. Lipsky, S. I. Korzhinsky, and B. A. Fedchenko, and their collections with over 300 specimens are held at the Komarov Botanical Institute herbarium (St. Petersburg). These collections formed the primary foundation for subsequent bryological studies in the region [8].

The first major taxonomic contributions to Central Asian bryology were made by V. F. Brotherus and G. Philibert, who described numerous taxa based on expeditionary materials and highlighted the exceptional floristic originality and high endemism rate within the region. Their work resulted in the description of several moss taxa that were new to science, including *Tortula grandiretis* Broth., *Tortula thianschanica* Broth., *Bryum pamirense* H. Philib. ex Broth., and *Bryum leptoglyphodon* H. Philib. A key milestone was the publication of the exsiccata series *Musci Turkestanici* [9], which included more than 100 bryophyte species from 15 families and provided the first systematic documentation of bryophyte diversity in Turkestan (Central Asian area including present-day Uzbekistan).

The pre-revolutionary period in the region culminated in the synthesis of *Flora Asiatskoi Rossii*, where Brotherus compiled the first regional overview of moss diversity across Asian Russia and Central Asia [8]. Brotherus’ work established the earliest taxonomic framework for bryophytes in the region and included some of the first published records from territories corresponding to modern Uzbekistan. Subsequent investigations in the Tien Shan and Pamir-Alay ranges led to the recognition of more than 50 species and the description of additional taxa that were new to science, including *Molendoa seravschanica* Broth. & Györffy, *Tortula submontana* Broth. (syn. *Syntrichia submontana* (Broth.) Ochyra), and *Tortula trachyphylla* Broth. [10,11]. These studies formed the taxonomic basis for all later bryological research all over Central Asia.

Following the establishment of Soviet scientific institutions, bryological research in Central Asia entered a phase of rapid expansion. During the first half of the twentieth century, floristic studies shifted from exploratory sampling to systematic regional inventories. A major contribution of this period was made by A. S. Lazarenko, whose revision of Pamir-Alay collections resulted in the publication of *Materials to the Bryoflora of Central Asia*, reporting 126 moss species and describing nine taxa that were new to science [12]. For several decades afterwards, this work remained a key reference for the identification of Central Asian mosses.

From the 1950s onwards, bryological research expanded significantly through extensive field surveys and taxonomic revisions across the Pamir-Alay, Tien Shan, Kopetdag, and Altai mountain systems. Important contributions were made by Rakhmatulina, Godvinsky, Akibaev, Abramova, Abramov, Kozlova, Tyvel, Yeremina, and Savicz-Lyubitskaya, who substantially improved knowledge of species distribution, ecology, and taxonomy in the region [13]. This period also witnessed the description of several taxa that were new to science, including genus *Usmania* Laz. (=*Campylostelium* Bruch & Schimp.), and species *Weissia papillosissima* Laz. (=*Weissia armata* (Thér. & Trab.) Fedosov), *Leiocolea mamatkulovii* (Duda) Duda (=*Mesoptychia mamatkulovii* (Duda) L. Söderstr. & Váňa) and *Fissidens karataviensis* Samsel [14,15,16], confirming Central Asia as a significant center of bryophyte diversity and endemism.

A particularly important contribution was made by U. K. Mamatkulov, whose studies from the 1960s to the 1990s addressed the taxonomy, ecology, phytogeography, and evolutionary history of Central Asian bryophytes [13]. His work documented approximately 320 species in the Pamir-Alay region and nearly 600 species across Central Asia, representing the most comprehensive synthesis of regional bryophyte diversity of that period. Despite substantial progress in neighboring countries, bryological research was unevenly distributed across Central Asia. While Tajikistan, Kazakhstan, and Kyrgyzstan were relatively well studied in view of other Central Asian areas, Uzbekistan remained comparatively underexplored, with most records derived from scattered floristic reports and herbarium-based observations.

The first systematic study in present-day Uzbekistan bryophyte flora was conducted in the Zarafshan Basin by O. F. Gaze, who recorded 47 bryophyte species and provided the earliest regional account of bryophyte diversity in the country [17]. Later, E. K. Rakhmatulina reported about 50 moss species for the Western Tien Shan, which became an important document about the mountain bryophytes of Uzbekistan [18,19]. Additional records were provided by A. M. Muzafarov, who reported bryophyte taxa from aquatic and semi-aquatic ecosystems of Uzbekistan [20]. Finally, Townsend [21,22,23] reported a limited number of bryophyte species, but his studies were of considerable significance because they provided critically evaluated and reliable records of rare and biogeographically important taxa, thereby contributing substantially to the understanding of bryophyte diversity in Uzbekistan.

A new phase of research began at the beginning of the 21st century with the revision of historical herbarium collections, in which M. S. Ignatov and E. A. Ignatova identified approximately 40 moss species from Uzbekistan and, based on specimens from the Zamin State Reserve, recorded *Brachythecium buchananii* (Hook.) A. Jaeger, as a new record for Central Asia [24]. This was followed by the Checklist of Mosses of East Europe and North Asia, which listed 103 moss species for Uzbekistan and provided the first synthesis and modern survey of national bryophyte diversity [25]. However, this checklist was largely based on historical records and thus outdated. Meanwhile, extensive nomenclatural changes in recent bryology science occurred, and comprehensive nation-wide field surveys with new moss records increased the need to prepare the new list of moss flora of Uzbekistan and its regions.

In recent years, bryophyte research in Uzbekistan has gradually expanded, but remains geographically limited. Dozens of studies of local flora have appeared over the last decade, but these studies only cover a fraction of the rather high ecological diversity [26].

Consequently, compared with other Central Asian countries, Uzbekistan still lacks a comprehensive and critically revised synthesis of its bryophyte flora. Existing data remain fragmented across the historical literature, regional floristic studies, and herbarium collections, while many areas remain insufficiently investigated. Critical aspects such as species distribution, ecological specialization, endemism, nomenclature, and conservation status have not yet been integrated into a unified national framework. Therefore, the development of an updated and critically revised checklist of Bryophyta for Uzbekistan is essential for advancing floristic, taxonomic, ecological, and conservation research in the region.

### 2.2. Checklist of Mosses of Uzbekistan

The new list of mosses in Uzbekistan not only provides a recent survey of Uzbek moss flora, but it is the first such list to bring exact records in administrative units of Uzbekistan. In total, 241 taxa of mosses are known to the country (234 species, 1 subspecies, 5 varieties, and 1 form), and among them 43 species were recently added to the national list of species. The list is given in Table 1. Species are listed in alphabetical order.

The results presented here clearly demonstrate that knowledge on moss flora and diversity within the country increased in recent decades. The results also showed the uneven research on the Uzbekistan territory, both spatially and temporary. In many areas, even whole administrative units remained obscured regarding local bryophyte flora knowledge, suggesting urgent needs for field work investigations, but also studies in other areas of bryophyte sciences. Even the best-investigated areas like Tashkent, Samarkand, Jizzakh, and Kashkadarya, although the richest in moss species, remained blurred, and new records are expected with further research. In contrast, the moss record analysis within the country highlight almost no records or severe neglect of bryophyte research in Namangan, Andijan, Navoi, Khorezm, and Karakalpakstan, which remained markedly underexplored. A clear temporal shift is also evident: while early records (pre-2000) dominate in certain regions, post-2001 data contribute substantially to the majority of recent floristic additions, particularly in central, eastern and southern parts of the country.

It is also important to emphasize that in recent years, the bryoflora of several administrative regions of Uzbekistan, including the Samarkand, Jizzakh, and Kashkadarya provinces, has been investigated more accurately. More than 3000 herbarium specimens collected from these regions are currently preserved at the Herbarium of Samarkand State University (SAMDU).

Threshold was made at 2000, so the recent records are considered those reported in this century. The symbols in cells are related to the most recent one, but all referenced reported species are given.

## 3. Discussion

**Figure 1:**
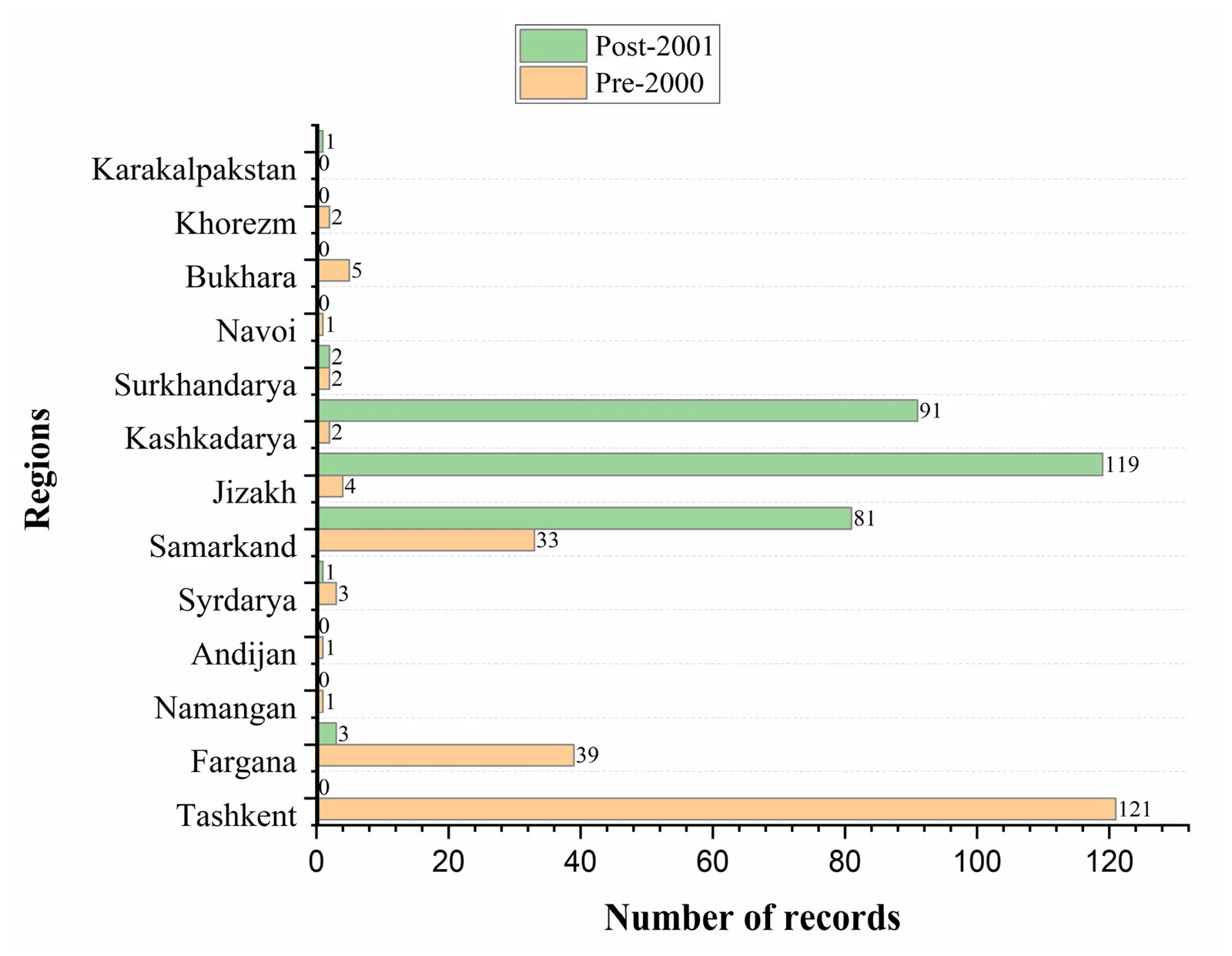
Spatio-temporal distribution of bryophyte taxa recorded across the administrative regions of Uzbekistan, grouped according to the period of their first records: up to 2000 and from 2001 onwards.

The spatio-temporal dynamics of bryological records across the administrative regions of Uzbekistan reflect historical shifts in floristic research intensity and collecting patterns (Figure 1). Prior to 2000, bryophyte documentation was highly localized, with records heavily concentrated in established botanical research centers, most notably in the Tashkent and Fergana regions, leaving much of the country under-sampled. Conversely, the period from 2001 onwards marks a considerable geographical expansion of bryological research into previously underexplored territories. Recent targeted collecting efforts have particularly focused on mountain ecosystems, resulting in a pronounced increase in new records from the Jizzakh, Kashkadarya, and Samarkand regions.

This contemporary trend indicates a transition from geographically localized collecting to more extensive exploration of natural habitats. However, the data presented in Figure 1 simultaneously highlight a considerable disparity in research coverage. While the number of documented records has increased substantially in several mountainous regions, some administrative regions, particularly the arid lowlands of Navoi, Bukhara, Khorezm, and Karakalpakstan, remain poorly investigated and bryophytes are considerably under-recorded. This uneven distribution of available data suggests that large parts of the country remain insufficiently investigated, emphasizing the need for expanded and strategically targeted floristic surveys to address historical gaps in bryological knowledge and develop a more comprehensive national inventory of bryophyte diversity with all local peculiarities, including species that are new to science. The high diversity differences documented are also used to acknowledge the possible influence of environmental conditions and habitat availability.

The results presented here can therefore guide the selection of insufficiently investigated areas for future studies, while also drawing attention to species that have not been recorded for long periods and/or may require further investigation in relation to the conservation and management of national bryophyte diversity, or species of unclear taxonomical status.

The highest numbers of documented taxa are recorded in the Tashkent, Samarkand, Jizzakh, and Kashkadarya regions, while several areas, including Namangan, Andijan, Navoi, Khorezm, and Karakalpakstan, remain markedly underexplored. *Fissidens karataviensis* has been recorded in Uzbekistan, but its precise locality remains unknown. A clear temporal shift is also evident: while records up to 2000 predominate in certain regions, records from 2001 onwards contribute substantially to recent floristic additions, particularly in the central and southern parts of the country. Considering that several dozen taxa are known from only a single documented record (the literature or specimen report) and that 78 of the 241 taxa documented from Uzbekistan have not been reported in the twenty-first century in any administrative unit, further field investigation of bryophytes throughout Uzbekistan remains necessary.

This spatial and temporal imbalance highlights persistent sampling bias and indicates that the bryophyte flora of Uzbekistan remains incompletely and unevenly investigated. Such heterogeneity underscores the necessity for targeted floristic surveys in poorly studied regions to achieve a more comprehensive understanding of national bryophyte diversity.

**Figure 2:**
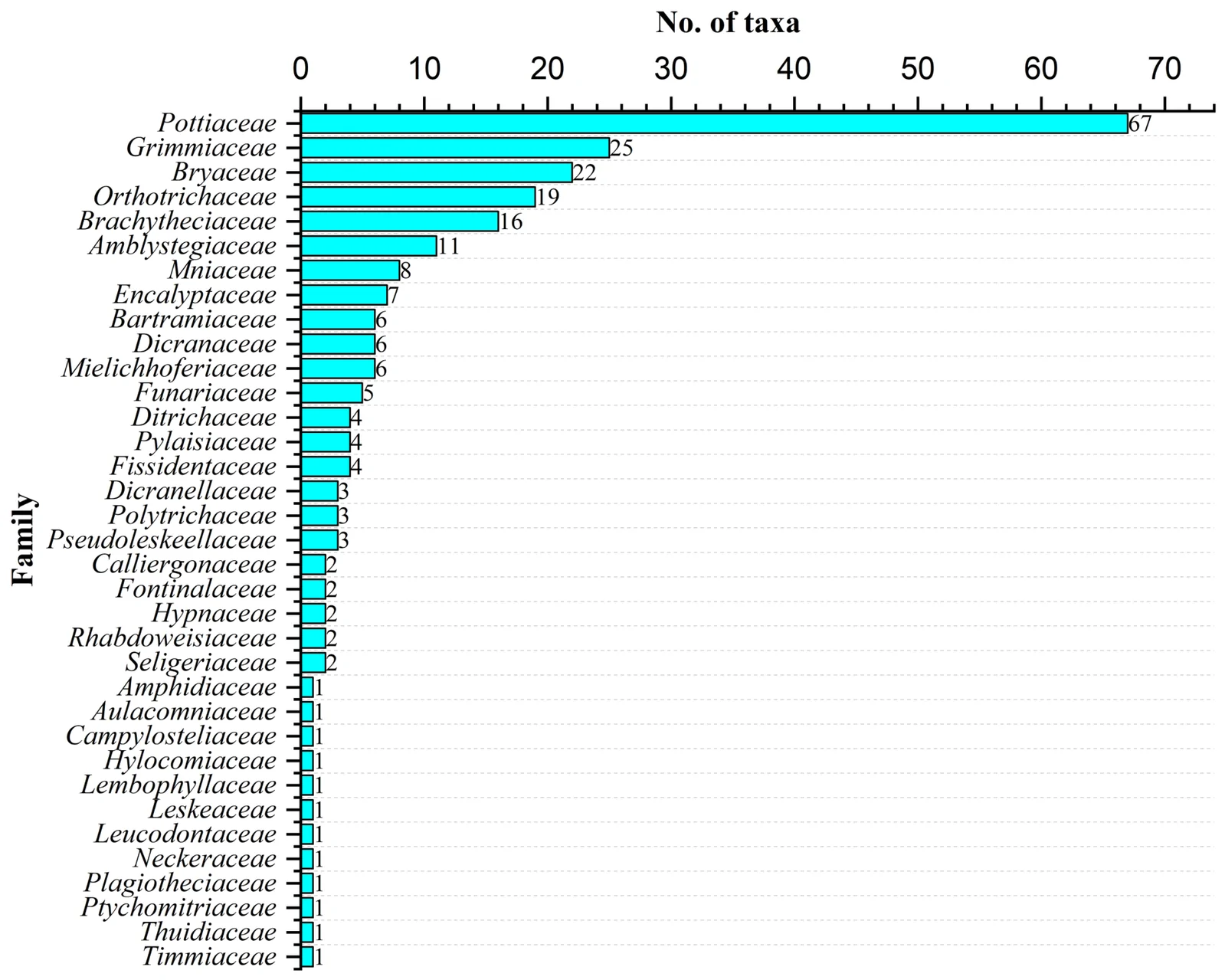
Distribution of moss taxa among families in Uzbekistan (a bar chart of species per family). Numbers at the ends of the bars indicate the number of taxa recorded in each family.

The taxonomic composition of the moss flora of Uzbekistan also shows considerable variation among families (Figure 2).

Pottiaceae is the most species-rich family, comprising 67 taxa (27.80% of the total flora), followed by Grimmiaceae with 25 taxa (10.37%), Bryaceae with 22 taxa (9.13%), Orthotrichaceae with 19 taxa (7.88%), and Brachytheciaceae with 16 taxa (6.64%). Amblystegiaceae is represented by 11 taxa (4.56%). Together, the five most species-rich families comprise 149 taxa, representing approximately 61.83% of the 241 taxa currently documented in Uzbekistan. In contrast, numerous families are represented by only one or a few taxa, demonstrating an uneven distribution of taxonomic richness among families, but also emphasizing aridity and xerotolerance among species recorded. Thus, pottiaceous mosses, for example, are widely taken to be the most represented in harsh environment, due their ability and plethora of strategies to survive prolonged drought.

The particularly high taxonomic richness of Pottiaceae, together with the substantial representation of Grimmiaceae, Bryaceae, Orthotrichaceae, and Brachytheciaceae, represents an important feature of the taxonomic structure of the Uzbek moss flora, and a biological spectra highlighting xero-tolerance. Further comparison with neighboring Central Asian bryofloras may help determine whether this family-level pattern is characteristic of Uzbekistan or reflects broader regional floristic and ecological patterns.

The taxonomic structure of the Uzbek moss flora should also be considered in relation to its biogeographical composition and the historical continuity of individual records. The present checklist integrates more than a century of bryological information, ranging from nineteenth- and early twentieth-century collections to recent field surveys. Consequently, it provides an opportunity not only to summarize the currently documented taxonomic richness of the flora, but also to reassess historically recognized endemic and geographically restricted elements in light of current taxonomy and distributional knowledge.

Endemism represents a particularly important component of the Central Asian bryoflora. Historical assessments recognized a considerable number of bryophytes as endemic to Central Asia; Mamatkulov and co-authors distinguished 52 endemic species within the Central Asian range type, whereas subsequent analyses demonstrated that the status of many historically recognized endemics required reassessment as taxonomic concepts and distributional knowledge expanded [8,51]. Some taxa formerly considered endemic to Central Asia have subsequently been recorded outside the region, whereas others have been reduced to synonymy with more widely distributed species. Historical endemic status should therefore not be transferred uncritically to the present flora.

Of particular biogeographical importance are those taxa that were historically regarded as Central Asian endemics and whose known distributions appear to remain restricted to the region according to currently available taxonomic and distributional evidence. At least six such taxa occur in Uzbekistan: *Fissidens karataviensis*, *Bryum leptoglyphodon*, *Tortula thianschanica*, *T. trachyphylla*, *T. transcaspica*, and *T. revolutifolia* [7,8,13,25,51,52]. These six taxa (taxonomic entries at different ranks) represent approximately 2.49% of the 241 taxa currently documented in Uzbekistan. They should, however, be regarded as Central Asian endemics occurring in Uzbekistan rather than as endemics of Uzbekistan, because their documented distributions extend beyond the political boundaries of the country.

The distinction between regional and national endemism is particularly important in Uzbekistan because the principal mountain systems supporting much of the country’s bryophyte diversity—the Western Tian Shan and Pamir-Alai—extend across national borders. Consequently, the occurrence of a type locality or an early historical collection within present-day Uzbekistan does not itself demonstrate national endemism. For example, the historical material of *Tortula trachyphylla* originates from the territory of present-day Uzbekistan, yet the species is also documented from other parts of Central Asia. Identification of strict national endemics therefore requires evidence that the currently accepted taxon is restricted to Uzbekistan rather than merely having been originally described as being from the country.

The occurrence of Central Asian endemics within the Uzbek moss flora is also relevant from a conservation perspective. Endemism itself does not imply that a species is threatened; nevertheless, geographically restricted taxa may warrant particular attention because their populations are confined to a comparatively limited regional range. Earlier Central Asian bryological assessments recognized the relationship between rarity, restricted distribution, and conservation relevance and emphasized the need for conservation attention, particularly for rare and endemic bryophytes [8,51,52]. Some taxa identified above as retaining Central Asian endemic distributions, including *Fissidens karataviensis* and *Tortula thianschanica*, were also recognized as rare or conservation-relevant elements of the regional bryoflora [52]. Thus, regional endemism should not be interpreted as evidence of threat per se; rather, when restricted distribution is accompanied by few known localities, predominantly historical records, or a lack of recent confirmation, such taxa become particularly important candidates for targeted field reassessment and subsequent conservation evaluation (otherwise they remained forgotten or as taxa with insufficient information).

This conservation perspective is supported by national practices elsewhere in Central Asia, where selected bryophytes have been incorporated into the Red Data Books, i.e., in Kazakhstan, Tajikistan, and Turkmenistan [53,54,55]. Of particular relevance is *Fissidens karataviensis*, which occurs in Uzbekistan and has also been recognized as a threatened bryophyte in neighboring Tajikistan [54]. Such national assessments cannot be transferred directly to Uzbekistan, because threat status depends on population size and trends, distribution, habitat condition, and threats within the country concerned. They nevertheless demonstrate the relevance of country-specific conservation assessment for transboundary endemic and geographically restricted bryophytes.

In contrast to these regional examples, bryophytes have not yet been represented in the national Red Data Book of Uzbekistan [56,57,58,59,60]. This absence should not be interpreted as evidence that the Uzbek moss flora lacks taxa of conservation concern. Rather, it should be considered in the context of historically uneven and still incomplete bryological knowledge in the country. The present checklist reveals substantial geographical and temporal gaps in the available records, while a number of taxa are known only from historical collections or a limited number of localities. Under these circumstances, assigning national threat categories solely on the basis of existing occurrence records would be premature. Instead, the checklist provides a framework for identifying priority taxa and localities for targeted reassessment.

Potential threats to the bryophyte flora of Uzbekistan should primarily be considered in relation to habitat alteration, the strong dependence of bryophytes on specific microhabitats and species sensitivity to climate change. Among anthropogenic pressures, excessive and poorly regulated livestock grazing may be particularly important, especially in foothill and mountain ecosystems. Overgrazing can reduce vegetation cover, increase trampling and soil disturbance, accelerate erosion and nitrification and alter the structure and moisture conditions of bryophyte microhabitats. However, species-specific responses of bryophytes to these pressures remain insufficiently documented in Uzbekistan. Therefore, the relative importance of individual threats should be evaluated through targeted field studies rather than inferred solely from general patterns of ecosystem degradation.

This checklist establishes a vital scientific baseline for future research and the development of Uzbekistan’s first national Red List of mosses. Although current data are not yet sufficient to assign official IUCN threat categories to most taxa, this document serves as an essential snapshot of existing bryophyte knowledge. To build a robust conservation framework, future efforts must prioritize Central Asian endemics, geographically restricted species, and taxa known only from historical records. Achieving this requires targeted field surveys to verify old localities, document population sizes, assess habitat conditions, and explore poorly investigated regions to close the remaining data gaps.

## 4. Materials and Methods

### 4.1. Data Collection and Literature Survey

The present study is based on an extensive review of the published literature, herbarium records, and available online biodiversity databases covering bryophyte diversity in Uzbekistan. The data was collected from several scientific sources, including international bibliographic databases such as Google Scholar, Scopus, and Web of Science, the Global Biodiversity Information Facility (GBIF) [37], specialized biodiversity platforms such as ResearchGate, and a review of existing libraries within the country irrespective of the publication language (including local and non-widely spoken languages). In addition, regional journals, monographs, expedition reports, and the historical taxonomic literature from the late nineteenth century to the end of October 2026 were systematically reviewed.

All available records referring to mosses (Bryophyta) from the territory of Uzbekistan were critically evaluated. Particular attention was given to ensuring the reliability of distributional data, taxonomic identity, and publication validity. Duplicated records and doubtful citations were excluded or marked accordingly in the final checklist.

### 4.2. Taxonomic Concept and Nomenclature Standardization

To ensure nomenclatural consistency, all taxa were verified and updated in accordance with the International Code of Nomenclature for algae, fungi, and plants [61]. Scientific names and their bibliographic citations were cross-checked against the World Flora Online (WFO) database [62], the International Plant Names Index [63], and the Tropicos database [64]. The Bryophyte Nomenclator [65] served as the primary complementary source for resolving synonymy, taxonomic ambiguities, and historical name changes specific to mosses. Additionally, geographic distribution records and occurrence data were validated using the Global Biodiversity Information Facility [37]. All species names reported in the literature were cross-checked against these comprehensive databases, and accepted names were standardized accordingly. Synonyms encountered in historical and regional publications were systematically compiled and are provided in the Appendix A of this article.

### 4.3. Geographic Scope of the Study

The present study is geographically confined to the territory of the Republic of Uzbekistan, a critical Central Asian biodiversity hotspot encompassing a highly heterogeneous range of ecological environments, from arid desert lowlands to alpine mountain ecosystems. For biogeographical and macro-analytical contexts, Uzbekistan is integrated within the broader Central Asian region. Nevertheless, all occurrence records documented in this checklist are strictly derived from specimens and observations verified within the internationally recognized political boundaries of the country.

**Figure 3:**
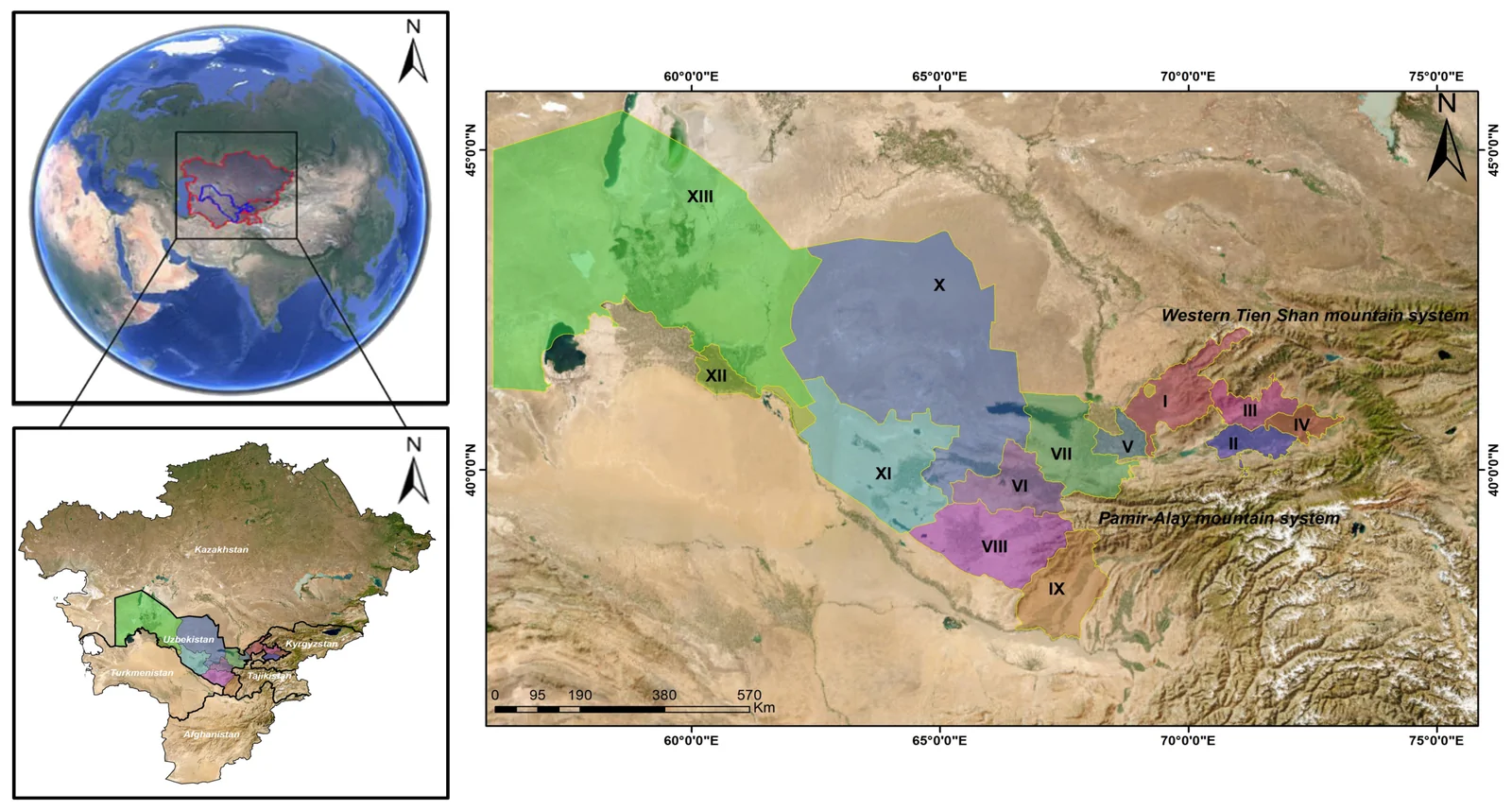
The position of Uzbekistan in the world, Central Asia, and its administrative units (I—Tashkent, II—Fargana, III—Namangan, IV—Andijan, V—Syrdarya, VI—Samarkand, VII—Jizakh, VIII—Kashkadarya, IX—Surkhandarya, X—Navoi, XI—Bukhara, XII—Khorezm, XIII—Karakalpakstan).

While the vast majority of the Uzbek territory is dominated by arid landscapes and extensive lowland plains, its environmental heterogeneity and species richness are profoundly enhanced by two major mountain systems. As explicitly illustrated in Figure 3, where these prominent systems are directly labeled with their respective names across the ranges, they comprise: (1) the Western Tian Shan Mountain System, which encompasses the Chatkal, Kurama, Pskem, Ugam, Karzhantau, and Maydantal mountain ranges, and (2) the Pamir-Alay Mountain System, which includes the Turkestan, Zarafshan, Hissar, Baysun, Kuhitang, Nuratau, and Molguzar mountain ranges. These mountain networks serve as vital refugia and centers of endemism, substantially driving the country’s unique biodiversity patterns.

The strategic positioning of these mountain systems within Uzbekistan establishes pronounced altitudinal, climatic, and ecological gradients, thereby generating a highly diverse mosaic of microhabitats that sustain a rich and remarkable bryophyte flora. For spatial modeling and macro-analytical precision, the administrative regions of Uzbekistan are designated by specific numerical codes, directly aligning with the spatial distribution records categorized in Table 1.

### 4.4. Inclusion Criteria and Data Treatment

To ensure scientific rigor, only occurrence data derived from peer-reviewed scientific publications, verified herbarium specimens, or authenticated database entries were integrated into the checklist. Conversely, any records lacking precise locality coordinates or robust taxonomic confirmation were treated as ambiguous and meticulously excluded from the final species census. In cases where multiple distinct sources documented the same taxon under conflicting names, synonymy was critically resolved in accordance with current globally accepted nomenclature, specifically leveraging The Bryophyte Nomenclator and World Flora Online. Intraspecific taxa are mentioned as such in cases where they are not typical, with the aim not to lose information which can be further studied.

### 4.5. Data Presentation

All taxonomically verified taxa were organized into a standardized, comprehensive checklist format. For each individual species, comprehensive metadata regarding publication histories, nomenclatural synonymy, and detailed phytogeographical distribution notes were structured within a comprehensive matrix (Table 1). The specific analytical symbols and notations employed throughout the checklist are explicitly defined immediately below the table.

## 5. Conclusions

The results of the present study demonstrate that Uzbekistan remains bryologically under-investigated and that its moss diversity is still insufficiently documented. The 241 taxa currently documented from the country should not be regarded as a final estimate of its moss diversity, and this number is expected to increase with further field investigation. Additional taxa may be discovered both as new records for Uzbekistan and, potentially, as species that are new to science. Further progress will require intensive field surveys, critical revision of herbarium collections, and, where appropriate, molecular approaches to resolve taxonomically problematic material. Nevertheless, the identity of some historically reported taxa may remain uncertain where original specimens are unavailable or insufficient for reliable taxonomic reassessment.

Although bryological research in Uzbekistan has intensified in recent years, substantial geographical and temporal gaps remain. Even comparatively well-documented regions such as Tashkent, Samarkand, Jizzakh, and Kashkadarya require further investigation, while poorly explored regions represent particularly important priorities for future surveys. The present study also highlights the biogeographical and conservation importance of Central Asian endemic, geographically restricted, and historically insufficiently documented taxa occurring in Uzbekistan. Their current status requires targeted field reassessment rather than inference from historical occurrence data alone. The present checklist therefore provides a critically revised taxonomic and distributional baseline for future floristic, taxonomic, ecological, and biogeographical research and for subsequent evidence-based conservation assessment using standardized criteria. Such assessments may ultimately provide the scientific basis for the development of a national Red List of mosses in Uzbekistan (threat categories not assessed in the present study).

## Figures and Tables

**Table 1:** Checklist of mosses in Uzbekistan. The following symbols are used throughout the checklist to indicate the origin, distribution, and verification status of records: ○—records published prior to 2000; ●—records published from 2001 onwards; *—species recorded for Uzbekistan *fide* Ignatov et al. [25]. **—species recently added (not previously known) to Uzbekistan moss flora. The regional subtotal is the sum across regions and does not necessarily correspond to the national total because some taxa occur in multiple regions.

U Z B E K I S T A N														
*Moss taxa* (Total 241)	Tashkent (I)	Fargana (II)	Namangan (III)	Andijan (IV)	Syrdarya (V)	Samarkand (VI)	Jizakh (VII)	Kashkadarya (VIII)	Surkhandarya (IX)	Navoi (X)	Bukhara (XI)	Khorezm (XII)	Karakalpakstan (XIII)	Reference
Regional subtotal	121	42	1	1	4	114	123	93	4	1	5	2	1	
*Amblystegium confervoides* (Brid.) Bruch, Schimp. & W. Guembel **							●							[26,27]
*Amblystegium serpens* (Hedw.) Schimp. *	○						●	●						[12,18,25,26,28,29]
*Amphidium lapponicum* (Hedw.) Schimp. *	○													[12,25]
*Anoectangium aestivum* (Hedw.) Mitt. **								●						[29]
*Atrichum tenellum* (Röhl.) Bruch & Schimp.	○													[19]
*Aulacomnium palustre* (Hedw.) Schwägr. **								●						[29]
*Barbula cylindrica* (Taylor) Schimp. f. *rufescens* C.E.O. Jensen						○								[17]
*Barbula unguiculata* Hedw. *						●	●	●						[17,26,29,30,31]
*Brachytheciastrum collinum* (Schleich. ex Müll.Hal.) Ignatov & Huttunen *	○							●						[12,20,25,32]
*Brachytheciastrum trachypodium* (Brid.) Ignatov & Huttunen *	○							●						[20,25,28,32]
*Brachytheciastrum velutinum* (Hedw.) Ignatov & Huttunen. **	○					●	●	●						[26,28,29,30,33]
*Brachythecium albicans* (Hedw.) Schimp.	○					●	●	●						[26,27,29,30,31,34]
*Brachythecium buchananii* (Hook.) A. Jaeger *						●	●	●						[24,25,26,29,30,31]
*Brachythecium gehebbi* Milde **							●							[26,27]
*Brachythecium mildeanum* (Schimp.) Schimp.ex Milde **							●							[26,27]
*Brachythecium plumosum* (Hedw.) Schimp. **						●	●							[26,27,33]
*Brachythecium rivulare* Schimp. *	○	○				●	●	●						[12,17,18,25,26,27,29,30,31,34]
*Brachythecium rutabulum* (Hedw.) Schimp.	○					●	●							[26,35,36,37]
*Brachythecium salebrosum* (Hoffm. ex F. Weber & D. Mohr) Schimp.						●	●	●						[17,26,29,30,31,33]
*Bryoerythrophyllum alpigenum* (Jur.) P. C. Chen	○													[28]
*Bryoerythrophyllum recurvirostrum* (Hedw.) P. C. Chen *	○					○								[18,25,37]
*Bryum argenteum* Hedw. *	○					●	●	●						[12,25,26,29,30,31]
*Bryum leptoglyphodon* H. Philib. *	○													[25,38]
*Bryum terskeiense* Paris.						●	●							[26,30,31]
*Buckia vaucheri* (Lesq.) D. Ríos, M.T. Gallego & J. Guerra		○												[21,37]
*Calcidicranella varia* (Hedw.) Bonfim Santos, Fedosov & Jan Kučera *	○					●		●						[12,18,25,26,29,30,31]
*Calliergonella cuspidata* (Hedw.) Loeske *	○													[12,18,25]
*Calliergonella lindbergii* (Mitt.) Hedenäs **							●							[26]
*Campylostelium pitardii* (Corb.) E. Maier *						○								[31,39]
*Ceratodon purpureus* (Hedw.) Brid. **							●	●						[26,40]
*Cinclidotus fontinaloides* (Hedw.) P. Beauv. *	○					○	●	●						[11,16,17,24,25,26,28]
*Cinclidotus riparius* (Host ex Brid.) Arn.	○													[19]
*Cratoneuron curvicaule* (Jur.) G. Roth.						●	●	●						[26,29,30,31]
*Cratoneuron filicinum* (Hedw.) Spruce *	○					●	●	●						[17,18,24,25,26,27,29,30,31,37]
*Crossidium squamiferum* (Viv.) Jur. *						●	●	●						[17,25,26,29,30,31,33]
*Crossidium squamiferum* var. *pottioideum* (De Not.) Mönk. *	○													[12,25]
*Cyrtomnium hymenophylloides* (Huebener) T.J. Kop. **							●							[26]
*Dicranella heteromalla* (Hedw.) Schimp.						●		●						[30,31,33]
*Dicranellopsis subulata* (Hedw.) Bonfim Santos, Siebel & Fedosov						●								[30,31]
*Dicranoweisia cirrata (Hedw.)* Lindb. ex Milde		○												[10]
*Dicranum brevifolium* (Lindb.) Lindb.						●								[30,31,33]
*Dicranum elongatum* Schleich. ex Schwägr.						●		●						[29,30,31,33]
*Dicranum muehlenbeckii* Bruch & Schimp.						●								[30,31]
*Dicranum polysetum* Swartz **							●							[26]
*Didymodon acutus* (Brid.) K. Saito *		○												[25,40]
*Didymodon icmadophilus* (Schimp. ex Müll. Hal.) K. Saito*	○	○						●						[25,37,40]
*Didymodon insulanus* (De Not.) M.O. Hill						●								[35]
*Didymodon rigidulus* Hedw. *		○					●	●						[26,29,40]
*Distichium capillaceum* (Hedw.) Bruch & Schimp. *	○	○			○	●	●	●			○			[10,12,18,25,26,28,29,30,31,37]
*Distichium inclinatum* (Hedw.) Bruch & Schimp. *		○				●	●	●						[17,25,26,29,30,31,41]
*Encalypta microstoma* Bals. -Criv. & De Not. **							●							[26,27]
*Encalypta pilifera* Funck. **								●						[29]
*Encalypta procera* Bruch						○								[37]
*Encalypta rhaptocarpa* Schwägr. *	○					●	●							[12,25,26,27,29,30,31]
*Encalypta spathulata* Müll.Hal.						○								[17]
*Encalypta streptocarpa* Hedw. *						●	●	●						[25,27,29,31]
*Encalypta vulgaris* Hedw. *	○					●	●	●						[12,17,25,26,27,29,30,31]
*Entosthodon muhlenbergii* (Turner) Fife. *	○						●							[12,25,26]
*Entosthodon pulchellus* (H. Philib.) Brugués								●						[29,32]
*Eucladium verticillatum* (With.) Bruch & Schimp.	○					○								[12,18,21]
*Eurhynchiastrum pulchellum* (Hedw.) Ignatov & Huttunen							○							[37]
*Eurhynchium savatieri* W.P. Schimper ex Bescherelle.						●	●	●						[26,29,30,31]
*Fissidens bryoides* Hedw. *							●	●						[26,27,29]
*Fissidens grandifrons* Brid. *	○	○				●	●	●						[17,18,25,26,29,30,31]
*Fissidens osmundoides* Hedw. **								●						[29]
*Fissidens karataviensis* Samsel														[25] not precise locality
*Fontinalis antipyretica* Hedw.	○					○	●	●						[17,18,26,29,31]
*Fontinalis hypnoides* C. J. Hartman. **							●	●						[26,29]
*Funaria hygrometrica* Hedw.						●	●	●						[17,26,27,29,30,31,33,36]
*Geheebia fallax* (Hedw.) R. H. Zander *		○												[25,40]
*Geheebia tophacea* (Brid.) R. H. Zander *	○					○	●				○			[18,25,26,37]
*Gemmabryum dichotomum* (Hedw.) J. R. Spence & H. P. Ramsay **							●	●						[27,29]
*Grimmia alpestris* (F. Weber & D. Mohr) Schleich.	○													[19,39]
*Grimmia anodon* Bruch & Schimp. *	○	○				●	●	●						[12,21,25,26,29,30,31,39]
*Grimmia caespiticia* (Brid.) Jur. *	○													[12,25]
*Grimmia capillata* De Not.						●								[30,31]
*Grimmia commutata* Huebener	○	○												[12,37]
*Grimmia crassifolia* Lindb. ex Broth. var. *cucullata* Baumg.		○												[21]
*Grimmia crinitoleucophaea* Cardot *	○	○												[12,25]
*Grimmia donniana* Sm.						●	●	●						[26,29,31]
*Grimmia elatior* Bruch ex Bals. -Criv. & De Not. **							●							[26,27]
*Grimmia laevigata* (Brid.) Brid. *	○					●	●	●						[12,25,26,29,30,31,39]
*Grimmia longirostris* Hook.						●	●	●						[26,29,30,31]
*Grimmia montana* Bruch & Schimp.						●	●	●						[26,29,30,31]
*Grimmia orbicularis* Bruch ex Wilson *	○	○				●		●						[21,25,29,30,31,37]
*Grimmia ovalis* (Hedw.) Lindb. *	○					○	●	●						[17,25,26,27,29]
*Grimmia pulvinata* (Hedw.) Sm. *	○					●	●	●						[12,25,26,27,29,30,31,39]
*Grimmia reflexidens* Müll. Hal *	○						●							[12,25,26]
*Grimmia tergestina* Tomm. ex Bruch & Schimp. *		○						●						[21,25,29]
*Gymnostomum aeruginosum* Sm. *	○													[18,21,25]
*Gymnostomum calcareum* Nees & Hornsch. *	○	○				●		●						[17,18,21,25,29,30,31]
*Haplodontium himalayanum* (Mitt.) Xiao R. Wang & J.C. Zhao						○								[34,42,43]
*Haplodontium macrocarpum* (Drumm.) J.R. Spence						○								[34,42]
*Homalothecium philippeanum* (Spruce) Schimp. *	○													[25,28]
*Hydrogonium bolleanum* (Müll.Hal.) A. Jaeger *		○												[25,37]
*Hydrogonium ehrenbergii* (Lorentz) A. Jaeger		○				○								[37,44]
*Hygroamblystegium humile* (P. Beauv.) Vanderp., Hedenäs & Goffinet	○													[37]
*Hygroamblystegium tenax* (Hedw.) Jenn						●	●	●						[26,29,31,32]
*Hygroamblystegium varium* (Hedw.) Mönk.						●								[45]
*Hygrohypnum luridum* (Hedw.) Jenn. *	○					●	●	●						[18,25,29,30,31]
*Hymenoloma crispulum* (Hedw.) Ochyra		○												[37]
*Hymenostylium recurvirostrum* (Hedw.) Dixon *	○	○				○								[12,17,18,25]
*Hypnum cupressiforme* Hedw. **	○						●							[26,28]
*Hypnum plumaeforme* Wilson. **							●							[26]
*Imbribryum alpinum* (Huds. ex With.) N. Pedersen	○													[37]
*Isothecium alopecuroides* (Lam. ex Dubois) Isov.						●	●	●						[26,29,30,31,33]
*Leptobryum pyriforme* (Hedw.) Wilson *	○							●						[12,18,25,29]
*Leptodictyum riparium* (Hedw.) Warnst. **							●							[26,27]
*Lescuraea radicosa* (Mitt.) Monk. **							●	●						[26,29]
*Leucodon sciuroides* (Hedw.) Schwägr. **	○													[19,28]
*Lewinskya affinis* (Brid.) F. Lara, Garilleti & Goffinet						●								[31,32]
*Lewinskya elegans* (Schwägr. ex Hook. & Grev.) F. Lara, Garilleti & Goffinet	○							●						[20,28,32]
*Lewinskya fastigiata* (Bruch ex Brid.) Vigalondo, F. Lara & Garilleti	○													[19]
*Lewinskya killiasii* (Müll. Hal.) Kiebacher, Kökinger & Yan Kučera	○													[19]
*Lewinskya rupestris* (Schleich. ex Schwägr.) F. Lara, Garilleti & Goffinet	○													[12]
*Lewinskya speciosa* (Nees) F. Lara, Garilleti & Goffinet	○													[19]
*Mnium lycopodioides* Schwägr. *						●								[25,30]
*Mnium marginatum* (Dicks.) P. Beauv.	○													[12]
*Mnium stellare* Hedw. **							●							[26,27]
*Mnium thomsonii* Schimp. *	○													[18,25]
*Molendoa seravschanica* Broth et Gyorff.						●		●						[17,29,30,31]
*Nyholmiella obtusifolia* (Brid.) Holmen & E. Warncke.	○					●	●							[19,26,28,30,31]
*Oncophorus virens* (Hedw.) Brid.						○		●						[10,17,29]
*Orthodicranum montanum* (Hedw.) Loeske *						○	●							[25,26]
*Orthotrichum anomalum* Hedw. *	○					●	●	●						[12,17,26,27,29,30,31]
*Orthotrichum crenulatum* Mitt.	○					●	●	●	●					[19,26,32,41,46,47]
*Orthotrichum cupulatum* Hoffm.ex Brid.		○				●	●	●						[17,21,25,26,27,29,30,31]
*Orthotrichum cupulatum* Hoffm. ex Brid. var. *riparium* Hueb.	○													[19]
*Orthotrichum hallii* Sull. & Lesq.						●								[31]
*Orthotrichum pallens* Bruch ex Brid. *							●	●						[25,26,27,29]
*Orthotrichum pamiricum* Plášek & Sawicki									●					[46]
*Orthotrichum patens* Bruch ex Brid.	○													[12,28]
*Orthotrichum pumilum* Sw.	○													[19,28]
*Orthotrichum schimperi* Hammar	○					○								[12,28,37]
*Orthotrichum stramineum* Hornsch. ex Brid. **	○													[19,28]
*Orthotrichum tenellum* Bruch ex Brid. *	○													[12,25,28]
*Oxyrrhynchium speciosum* (Brid.) Warnst. *	○													[18,19,25]
*Palustriella commutata* (Hedw.) Ochyra *	○	○	○			●	●	●						[12,17,18,20,25,26,29,30,31]
*Philonotis caespitosa* Jur. **								●						[29]
*Philonotis calcarea* (Bruch & Schimp.) Shimp. *	○	○		○										[12,18,25,37]
*Philonotis fontana* (Hedw.) Brid. *	○					●		●						[12,17,18,25,29,30,31]
*Philonotis marchica* (Hedw.) Brid. *	○													[18,19,21,25]
*Philonotis seriata* Mitt. *	○					●	●							[18,25,26,30,31]
*Philonotis tomentella* Molendo *	○													[12,18,21]
*Physcomitrium eurystomum* Sendtn. *	○													[18,19,25]
*Physcomitrium pyriforme* (Hedw.) Brid. *	○					○		●						[17,18,25,29]
*Plagiomnium acutum* (Lindb.) T.J. Kop. **						○								[17]
*Plagiomnium medium* (Bruch & Schimp.) T.J. Kop. *	○													[18,25]
*Plagiomnium medium curvatulum* T. J. Kop. (Lindb)						●								[31]
*Plagiothecium nemorale* Jaeger. **							●							[26]
*Platydictya jungermanniodes* (Brid.) H.A. Crum **							●							[26]
*Pleurozium schreberi* (Willd. ex Brid.) Mitt.						●								[30,31]
*Pohlia cruda* (Hedw.) Lind. *	○						●							[12,25,26,27]
*Pohlia elongata* Hedw.		○												[37]
*Pohlia melanodon* (Brid.) A.J. Shaw *	○													[18,19,25]
*Pohlia nutans* (Hedw.) Lindb.						●		●						[17,29,30,31]
*Pohlia wahlenbergii* (F. Weber & D. Mohr) A.L. Andrews *	○						●							[19,21,25,26,27]
*Pohlia wahlenbergii* var. *glacialis* (Schleich. ex Brid.) E.F. Warb.	○													[12]
*Polytrichum juniperinum* Hedw. *	○					●	●							[12,17,25,26,30,31]
*Polytrichum piliferum* Hedw. *	○													[8,25]
*Pseudocrossidium hornschuchianum* (Schultz) R.H. Zander	○						●							[19,48]
*Pseudoleskeella papillosa* (Lindb.) Kindb.						●		●						[26,29,30,31,33]
*Pseudoleskeella tectorum* (Funck ex Brid.) Kindb. ex Broth.	○	●					●	●						[20,26,27,28,32]
*Pseudoleskeella catenulata* (Brid. ex Schrad.) Kindb. **							●							[26]
*Pterygoneurum lamellatum* (Lindb.) Jur. *	○						●							[12,25,26,27]
*Pterygoneurum ovatum* Dixon *	○					○	●	●						[12,17,25,26,27,29]
*Pterygoneurum subsessile* (Brid.) Jur. *						●								[25,30,31,33]
*Ptychomitrium indicum* (Willd. ex Schrad.) A. Jaeger						○								[23,25]
*Ptychostomum bimum* (Schreb.) J.R. Spence						●	●							[17,26,27,30,31]
*Ptychostomum creberrimum* (Taylor) J.R. Spence & H.P. Ramsay **							●							[26,27]
*Ptychostomum funkii* (Schwägr.) J.R. Spence *	○					●		●						[12,25,29,30,31,41]
*Ptychostomum imbricatulum* (Müll. Hal.) Holyoak & N. Pedersen		○				●	●	●						[17,26,27,29,30,33]
*Ptychostomum lonchocaulon* (Müll.Hal.)						●								[30,31]
*Ptychostomum pallens* (Sw.) J.R. Spence **							●							[26]
*Ptychostomum pallescens* (Schleich. ex Schwägr.) J.R. Spence *	○						●							[12,25,26,27]
*Ptychostomum pseudotriquetrum* (Hedw.) J.R. Spence & H.P. Ramsay *	○	○				●	●	●						[12,25,26,27,29,31,41]
*Ptychostomum schleicheri* (DC.) J. R. Spence ex D. Bell & Holyoak *	○					●	●	●						[12,17,18,26,27,30,33]
*Ptychostomum turbinatum* (Hedw.) J. R. Spence *	○	●				○	●							[17,18,25,26,29]
*Racomitrium sudeticum* (Funck) Bruch & Schimp. **								●						[29]
*Rhynchostegium riparioides* (Hedw.) Cardot *	○					●	●	●						[17,18,25,26,27,29,30,31]
*Roaldia revoluta* (Mitt.) P.E.A.S. Câmara & Carv. -Silva *	○	○					○							[12,21,25,28,37]
*Rosulabryum capillare* (Hedw.) J. R. Spence	○													[37]
*Rosulabryum elegans* (Nees ex Brid.) Ochyra						●	●	●						[26,27,29,30,31]
*Rosulabryum flaccidum* (Brid.) J. R. Spence	○													[12]
*Rosulabryum moravicum* (Podp.) Ochyra & Stebel *								●						[20,25,32]
*Saelania glaucescens* (Hedw.) Broth.		○												[37]
*Schistidium agassizii Sull. & Lesq.*	○													[20]
*Schistidium apocarpum* (Hedw.) Bruch & Schimp.						●	●	●						[26,27,29,31]
*Schistidium brunnescens* Limpr. *	○													[12,25]
*Schistidium confertum* (Funck) Bruch & Schimp.	○													[19]
*Schistidium flaccidum* (De Not.) Ochyra *	○													[12,25]
*Schistidium platyphyllum* (Mitt.) Perss.	○													[19]
*Schistidium rivulare* (Brid.) Podp. *	○						●	●						[18,21,26,27,29]
*Scopelophila ligulata* Spruce **							●							[26]
*Seligeria donniana* (Sm.) Müll. Hal.						●								[31]
*Seligeria pusilla* (Hedw.) Bruch & Schimp.						●	●							[26,31]
*Streblotrichum convolutum* (Hedw.) P. Beauv. **							●							[26]
*Streblotrichum enderesii* (Garov.) Loeske *	○					●								[12,25,26,29,30]
*Syntrichia calcicola* Amann **							●	●						[26,29]
*Syntrichia caninervis* Mitt. *	○	○			○	●	●	●		○	○	○	●	[8,13,25,26,29,30,31,49]
*Syntrichia handelii* Sciffn. var. *ferganensis* (Laz.) Ochyra *		○			●	○								[12,25,49]
*Syntrichia handelii* S. Agnew & Vondr.	○	○					●	●						[27,28,29,37]
*Syntrichia laevipila* Brid.						●	●	●						[26,27,29,30,31,33]
*Syntrichia latifolia* (Bruch ex Hartm.) Hueb **							●	●						[26,29]
*Syntrichia norvegica* F. Weber *	○						●	●						[12,25,26,29]
*Syntrichia princeps* (De Not.) Mitt. *					○	●	●	●			○			[8,25,26,29,30,31,33,37]
*Syntrichia pseudohandelii* (J. Froehl.) S. Agnew & Vondr. *		●				○								[21,25,37,49]
*Syntrichia ruralis* Weber & D. Mohr. *	○	○				○	●	●						[8,17,27,28,29,49]
*Syntrichia ruralis* Weber & D. Mohr. var. *ruraliformis* (Besch.) Delogne **							●	●						[26,40]
*Syntrichia ruralis* Weber & D. Mohr. subsp. *hirsuta* (Venturi) Düll.	○													[12,28]
*Syntrichia virescens* (De Not.) Ochyra						●		●						[29,45]
*Thamnobryum alopecurum* (Hedw.) Nieuwl. ex Gangulee **							●							[26]
*Thuidium abietinum* (Hedw.) Schimp. **		○				○								[17,37]
*Timmia bavarica* Hessl *	○	○				○	●							[8,12,17,25,26,27]
*Tortella alpicola* Dixon *		○												[25,50]
*Tortella tortuosa* (Schrad. ex Hedw.) Limpr.	○													[12]
*Tortula acaulon* (With.) R.H. Zander *							●							[25,26,27]
*Tortula atrovirens* (Turner ex Sm.) Lindb. *							○							[13,25]
*Tortula brevissima* Schiffn. **							●							[26,27]
*Tortula cuneifolia* (Dicks.) Turner *						○								[13,25]
*Tortula grandiretis* Broth. *	○					○			○			○		[7,12,21,25]
*Tortula hoppeana* (Schultz) Ochyra	○						●							[12,26]
*Tortula inermis* (Brid.) Mont *	○					●	●	○						[12,20,25,26,27,30,31]
*Tortula mucronifolia* Schwägr.						●	●	●						[17,26,29,30,31]
*Tortula muralis* (L. ex Hedw.)	○					●	●	●						[12,25,26,27,29,30,31,33]
*Tortula obtusifolia* (Schwägr.) Mathieu *	○	○				●		●						[12,17,25,29,30,31]
*Tortula protobryoides* R.H. Zander	○													[19]
*Tortula revolutifolia* Laz. *	○					●								[12,25,30,31]
*Tortula schmidii* (Müll. Hal.) Broth. **							●							[26]
*Tortula subulata* Hedw. *	○					●	●	●						[17,25,26,27,29]
*Tortula thianschanica* Broth. *	○	○							○					[7,12,21,25]
*Tortula trachyphylla* Broth. *	○					●	○	○						[7,11,12,25,30,31]
*Tortula transcaspica* Broth. *	○													[21,25]
*Tortula vlassovii* (Laz.) Ros & Herrnst. *	○													[12,25]
*Trichostomopsis australasiae* (Hook. & Grev.) H. Rob. *		○				○								[22,25]
*Trichostomopsis umbrosa* (Müll. Hal.) H. Rob.											○			[40]
*Vinealobryum vineale* (Brid.) R.H. Zander.	○					○								[12,40]
*Warnstorfia exannulata* (Schimp.) Loeske						○								[17]
*Warnstorfia fluitans* (Hedw.) Loeske						○								[17]
*Weissia brachycarpa* Juratzka. **							●							[26]
*Weissia condensa* (Voit) Lindb. **							●	●						[26,29]

## Data Availability

The authors can provide additional data upon request.

## Appendix A

### List of Synonyms Found in the Literature Sources and the Treatment Followed in This Study

***Amblystegium serpens* (Hedw.) Schimp.** = *Amblystegium adpressum* (Velen.) Velen., *Amblystegium atrovirens* Hesselbo, *Amblystegium juratzkanum* Schimp., *Amblystegium serpens* var. *juratzkanum* (Schimp.) Rau & Herv., *Amblystegium serpens f.* affine (Loeske) Podp., *Hypnum serpens* Hedw., *Hypnum serpens var. juratzkanum* (Schimp.) Bonsd., *Leskea repens* Schleich. ex Schultz, *Stereodon serpens* (Hedw.) Brid.

***Atrichum tenellum* (Röhl.) Bruch & Schimp.** = *Atrichum crispum* var. *molle* (Holz.) Frye, *Atrichum tenellum* f. *affine* (Warnst.) Podp., *Atrichum tenellum* f. *majus* (H.A. Möller) Podp., *Atrichum tenellum* f. *obtusatum* (Latzel) Podp., *Atrichum tenellum* var. *cylindricum* (Podp.) Podp., *Atrichum undulatum* var. *minus* (Hedw.) Thér. & Mong., *Bryum marginatum* var. *orthorhynchum* (Brid.) Hartm., *Bryum orthorhynchum* (Brid.) Brid., *Catharinea mollis* Holz., *Catharinea tenella* Röhl., *Mnium orthorhynchum* Brid., *Oligotrichum undulatum* var. *minus* (Hedw.) DC., *Pogonatum undulatum* var. *minus* (Hedw.) Opiz, *Polla orthorhyncha* (Brid.) Brid., *Polytrichum undulatum* var. *tenellum* (Röhl.) Wahlenb.

***Brachytheciastrum trachypodium* (Brid.) Ignatov & Huttunen** = *Brachythecium payotianum* Schimp. ex Boulay, *Brachythecium trachypodium* (Brid.) Schimp., *Brachythecium trachypodium* var. *payotianum* (Schimp. ex Boulay) Mönk., *Brachythecium velutinum* f. *sericeum* (Brid.) Podp., *Brachythecium velutinum* subsp. *payotianum* (Schimp. ex Boulay) J.J. *Amann, Eurhynchium trachypodium* (Brid.) Kindb., *Hypnum sericeum* Funck, *Hypnum trachypodium* (Brid.) Müll.Hal., *Isothecium trachypodium* Brid.

***Brachythecium rivulare* Schimp.** = *Brachythecium permolle* Müll.Hal., *Brachythecium rivulare* f. *cataractarum* (Saut.) Mönk., *Brachythecium rivulare* var. *alare* (Dixon) Vohra, *Brachythecium rutabulum* var. *fallax* M.T. Lange, *Hypnum rivulare* (Schimp.) Bruch.

***Brachythecium salebrosum* (Hoffm. ex F. Weber & D. Mohr) Schimp.** = *Hypnum salebrosum* Hoffm. ex F. Weber & D. Mohr, *Hypnum thomasii* Brid., *Lescuraea affinis* (Limpr.) Broth.

***Brachytheciastrum trachypodium* (Brid.) Ignatov & Huttunen** = *Brachythecium trachypodium* (Brid.) Schimp., *Hypnum trachypodium* (Brid.) Müll.Hal.

***Bryoerythrophyllum aeneum* (Müll. Hal.) B.H. Allen** = *Bryoerythrophyllum recurvirostrum var. aeneum* (Müll.Hal.) R H Zander

***Bryum argenteum* Hedw.** = *Bryum argentatum* Müll.Hal.

***Ptychostomum pseudotriquetrum* (Hedw.) J.R. Spence & H.P. Ramsay*** = Bryum pseudotriquetrum* (Hedw.) Gaertn., *Bryum ventricosum* Dicks.

***Ptychostomum pallens* (Sw.) J.R. Spenc *=**** Bryum subelegans* Kindb., *Bryum acutiusculum* Müll.Hal.

***Ptychostomum pallescens* (Schleich. ex Schwägr.) J.R. Spence *=**** Bryum pallescens* Schleich. ex Schwägr., *Bryum obconicum* Hornsch.

***Calcidicranella varia* (Hedw.) Bonfim Santos, Fedosov & Jan Kučera *=**** Anisothecium varium* (Hedw.) Mitt., *Anisothecium varium* (Hedw.) Mitt., *Dicranella varia* (Hedw.) Schimp.

***Cinclidotus fontinaloides* (Hedw.) P. Beauv.*** = Cinclidotus fontinaloides* var. *aurosicus* Thér., *Cinclidotus fontinaloides* var. *baumgartneri* E. Bauer, *Cinclidotus minor* Lindb.

***Cinclidotus riparius* (Host ex Brid.) Arn.*** = Cinclidotus riparius* f. *brevifolius* H. Schmidt, *Gymnostomum riparium* Host ex Brid., *Cinclidotus nigricans* (Brid.) Dixon.

***Cratoneuron filicinum* (Hedw.) Spruce*** = Amblystegium campyliopsis* Dixon, *Cratoneuron filicinum* var. *aciculinum* (Müll.Hal. & Kindb.) Grout, *Hygroamblystegium filicinum* (Hedw.) Loeske, *Hypnum fallax* Brid., *Thuidium filicinum* (Hedw.) Kindb.

***Crossidium squamiferum* (Viv.) Jur.*** = Barbula membranifolia* (Hook.) Schultz, *Crossidium squamiferum* var. *longipilum* J.J. Amann, *Tortula squamifera* (Viv.) De Not.

***Calcidicranella varia* (Hedw.) Bonfim Santos, Fedosov & Jan Kučera *=**** Dicranella varia* (Hedw.) Schimp., *Anisothecium varium* (Hedw.) Mitt.

***Distichium capillaceum* (Hedw.) Bruch & Schimp.*** = Distichium capillaceum* var. *brevifolium* Bruch & Schimp., *Distichium capillaceum* f. *brevifolium* Roiv., *Distichium montanum* I. Hagen.

***Distichium inclinatum* (Hedw.) Bruch & Schimp.*** = Distichium inclinatum* f. *elongatum* (J.J. Amann) Podp., *Swartzia inclinata* (Hedw.) P. Beauv.

***Encalypta vulgaris* Hedw.*** = Encalypta extinctoria* Sw. ex Lag., *Encalypta vulgaris* var. *apiculata* Wahlenb.

***Eucladium verticillatum* (With.) Bruch & Schimp.*** = Eucladium verticillatum* var. *acuminatum* Głow.

***Fissidens grandifrons* Brid.*** = Fissidens grandifrons* var. *planicaulis* (Besch.) Nog.

***Fontinalis antipyretica* Hedw.*** = Fontinalis antipyretica* f. *alpestris* (Milde) Mönk.

***Funaria hygrometrica* Hedw.*** = Mnium hygrometricum* (Hedw.) With., *Funaria hygrometrica* var. *apiculata* Vilh.

***Geheebia fallax* (Hedw.) R.H. Zander*** = Barbula unguiculata* Hedw., *Barbula unguiculata* var. *acuminata* (Hedw.) Brid.

***Geheebia tophacea* (Brid.) R.H. Zander*** = Didymodon tophaceus* (Brid.) Lisa

***Grimmia anodon* Bruch & Schimp.*** = Grimmia moxleyi* R.S. Williams

***Grimmia crinita* Brid.*** = Grimmia crinita f. brevis* (Boulay) Podp.

***Grimmia laevigata* (Brid.) Brid.*** = Grimmia campestris* Burch. ex Hook.

***Grimmia ovalis* (Hedw.) Lindb.*** = Grimmia commutata* Huebener.

***Gymnostomum aeruginosum* Sm.*** = Gymnostomum aeruginosum* f. *compactum* (Bruch & Schimp.) Margad.

***Gymnostomum calcareum Nees & Hornsch.**** = Gymnostomum calcareum* var. *australe* Broth. & Geh., *Mollia calcarea* (Nees & Hornsch.) Lindb.

***Haplodontium macrocarpum* (Drumm.) J.R. Spence*** = Mielichhoferia macrocarpa* (Drumm.) Bruch & Schimp.

***Homalothecium philippeanum* (Spruce) Schimp.*** = Homalothecium philippeanum* f. *densum* (De Not.) Podp.

***Hygrohypnum luridum* (Hedw.) Jenn.*** = Hygrohypnum luridum* f. *alpinum* (J.J. Amann) Podp.

***Hymenostylium recurvirostrum* (Hedw.) Dixon*** = Hymenostylium curvirostrum* Mitt., *Hymenostylium recurvirostrum* var. *anoectangioides* (Thér.) Wijk & Margad.

***Hypnum cupressiforme* Hedw.*** = Hypnum cupressiforme* var. *aduncoides* Brid.

***Leptobryum pyriforme* (Hedw.) Wilson*** = Leptobryum pyriforme* f. *appendiculatum* (Warnst.) Podp.

***Leucodon immersus* Lindb.*** = Leucodon sciuroides* var. *antitrichioides* Plutenko.

***Lewinskya elegans* (Schwägr. ex Hook. & Grev.) F. Lara, Garilleti & Goffinet*** = Dorcadion elegans* (Schwägr. ex Hook. & Grev.) Lindb., *Orthotrichum elegans* Schwägr. ex Hook. & Grev., *Orthotrichum speciosum* subsp. *elegans* (Schwägr. ex Hook. & Grev.) Lindb., *Orthotrichum speciosum* var. *elegans* (Schwägr. ex Hook. & Grev.) Warnst.

***Lewinskya fastigiata* (Bruch ex Brid.) Vigalondo, F. Lara & Garilleti*** = Orthotrichum fastigiatum* Bruch ex Brid.

***Lewinskya rupestris* (Schleich. ex Schwägr.) F. Lara, Garilleti & Goffinet*** = Orthotrichum rupestre* Schleich. ex Schwägr., *Orthotrichum rupestre* var. *sturmii* (Hoppe & Hornsch.) Jur., *Orthotrichum sturmii* Hoppe & Hornsch.

***Microbryum vlassovii* (Laz.) Zander*** = Lydiaea vlassovii* (Laz.) Laz., *Microbryum vlassovii* (Laz.) R.H. Zander, *Phascum vlassovii* Laz.

***Mnium marginatum* (With.) Brid. ex P. Beauv.** *= Mnium riparium* Mitt.

***Molendoa seravschanica* Broth. & Györffy.**

***Nyholmiella obtusifolia* (Brid.) Holmen & E. Warncke*** = Stroemia obtusifolia* (Brid.) I. Hagen.

***Oncophorus virens* (Hedw.) Brid.*** = Cynodontium virens* (Hedw.) Schimp., *Aongstroemia bicolor* Müll.Hal., *Oncophorus bicolor* (Paris) Broth.

***Orthotrichum alpestre* Hornsch. ex Bruch & Schimp.*** = Orthotrichum patens subsp. alpestre* (Hornsch. ex Bruch & Schimp.) Boulay.

***Orthotrichum anomalum* Hedw.*** = Dorcadion anomalum* (Hedw.) Lindb.

***Orthotrichum schimperi* Hammar*** = Dorcadion schimperi* (Hammar) Lindb., *Orthotrichum fallax* Schimp., *Orthotrichum pumilum* Bruch & Schimp., *Orthotrichum schimperi var. intermedium* Grönvall.

***Oxyrrhynchium speciosum* (Brid.) Warnst.*** = Oxyrrhynchium speciosum* f. *densum* (Warnst.) Podp.

***Palustriella commutata* (Hedw.) Ochyra*** = Cratoneuron glaucum* (DC.) Wheldon, *Cratoneuron commutatum* (Hedw.) Loeske, *Palustriella commutata* var. *irrigata* (Amann) Ochyra

***Philonotis fontana* (Hedw.) Brid.*** = Mnium fontanum* Hedw., *Philonotis fontana* f. *acutifolia* Papp, *Philonotis seriata* var. *adpressa* (Fergusson) Bryhn.

***Philonotis marchica* (Hedw.) Brid.*** = Philonotis marchica* var. *aristata* Renauld

***Philonotis tomentella* Molendo*** = Philonotis tomentella* var. *adpressa* Loeske

***Physcomitrium acuminatum* Bruch & Schimp.*** = Physcomitrium eurystomum* f. *acuminatum* (Bruch & Schimp.) Loeske.

***Physcomitrium pyriforme* (Hedw.) Brid.*** = Bryum pyriforme* (Hedw.) With., *Physcomitrium pyriforme* f. *carinatum* (Warnst.) Podp.

***Plagiomnium cuspidatum* (Hedw.) T.J. Kop.*** = Mnium cuspidatum* Hedw.

***Plagiomnium medium* (Bruch & Schimp.) T.J. Kop.*** = Mnium medium* Bruch & Schimp.

***Pohlia cruda* (Hedw.) Lindb.*** = Webera cruda* (Hedw.) Fürnr.

***Pohlia melanodon* (Brid.) A.J. Shaw*** = Mniobryum delicatulum* (Hedw.) Dixon

***Pohlia nutans* (Hedw.) Lindb.*** = Webera nutans* Hedw.

***Pohlia wahlenbergii* (Web. & Mohr) Andrews * =**** Mniobryum albicans* (Wahlenb.) Limpr.

***Pohlia wahlenbergii* var. *glacialis* (Brid.) Warb.*** = Bryum albicans* var. *glaciale* (Schleich. ex Brid.) Müll.Hal., *Mniobryum albicans* var. *glaciale* (Schleich. ex Brid.) Limpr., *Mniobryum wahlenbergii var. glaciale* (Schleich. ex Brid.) Wijk & Margad.

***Polytrichum juniperinum* Hedw**. * = Polytrichum juniperinum var. alpinum* Schimp.

***Pseudoleskeella tectorum* (Funck ex Brid.) Kindb. ex Broth. =*** Pseudoleskeella tectorum* subsp. *cyrtophylla* (Kindb.) Lewinsky.

***Pterygoneurum ovatum* (Hedw.) Dixon*** = Bryum ovatum* (Hedw.) Dicks., *Pterygoneurum pusillum* C.E.O. Jensen.

***Ptychostomum imbricatulum* (Müll. Hal.) Holyoak & N. Pedersen** = *Bryum caespiticium* Hedw.

***Ptychostomum pseudotriquetrum* (Hedw.) J.R. Spence & H.P. Ramsay*** = Bryum ventricosum* Dicks., *Mnium pseudotriquetrum* Hedw.

***Ptychostomum schleicheri* (DC.) J.R. Spence*** = Bryum schleicheri* DC.

***Ptychostomum schleicheri* var. *latifolium* (Schwägr.) D. Bell & Holyoak*** = Bryum latifolium* (Schwägr.) Brid.

***Ptychostomum turbinatum* (Hedw.) J.R. Spence*** = Bryum turbinatum* (Hedw.) Turner.

***Rhynchostegium riparioides* (Hedw.) Cardot*** = Rhynchostegium riparioides, Rhynchostegium rusciforme* Schimp., *Rhynchostegium rusciforme* var. *laminatum* (Boulay) Réchin & Sebille.

***Roaldia revoluta* (Mitt.) P.E.A.S. Câmara & Carv. -Silva** = *Hypnum revolutum* (Mitt.) Lindb.

***Schistidium brunnescens* Limpr.*** = Schistidium brunnescens* f. *epilosum* (Schiffn.) Vilh.

***Schistidium rivulare* (Brid.) Podp.*** = Schistidium alpicola* var. *rivulare* (Brid.) Limpr.

***Schistidium flaccidum* (De Not.) Ochyra*** = Grimmia flaccida* (De Not.) Lindb.

***Syntrichia caninervis* Mitt.*** = Syntrichia desertorum* (Broth.) J.J. AmannTortula desertorum Broth.

***Syntrichia caninervis* var. *abranchesii* (Luisier) R.H. Zander*** = Syntrichia montana* subsp. *abranchesii* (Luisier) Podp.

***Syntrichia ruralis* (Hedw.) F. Weber & D. Mohr*** = Tortula ruralis* (Hedw.) G. Gaertn., B. Mey. & Scherb., *Barbula ruralis* Hedw.

***Syntrichia ruralis* subsp. *hirsuta* (Venturi) Düll** = *Syntrichia papillosissima* (Copp.) Loeske.

***Syntrichia ruralis* var. *ruraliformis* (Besch.) Delogne*** = Syntrichia ruralis* var. *aciphylloides* (Podp.) Podp.

***Timmia megapolitana* Hedw.subsp. *bavarica*** (Hessl.) Brassard * = Timmia bavarica* Hessl.

***Tortula hoppeana* (Schultz) Ochyra*** = Desmatodon latifolius* (Hedw.) Brid.

***Tortula mucronifolia* Schwägr.*** = Syntrichia mucronifolia* (Schwägr.) Brid.

***Tortula obtusifolia* (Schwägr.) J. Kickx f.*** = Tortula obtusifolia* var. *brevifolia* (Schimp.) G. Roth.

***Tortula subulata* Hedw.*** = Syntrichia subulata* (Hedw.) F. Weber & D. Mohr.

***Vinealobryum insulanum* (De Not.) R.H. Zander*** = Barbula cylindrica* (Taylor) Schimp. ex Boulay, *Zygotrichia cylindrica* Taylor.

***Warnstorfia exannulata* (Schimp.) Loeske*** = Drepanocladus exannulatus* (Schimp.) Warnst., *Hypnum exannulatum* Schimp.
